# Impact of sleep apnea severity on left ventricular remodelling

**DOI:** 10.6026/973206300211593

**Published:** 2025-06-30

**Authors:** Vutukuri Kiranmai, Amoolya Rao Amaravadhi, Vaishnavi Jamched, Kosmita Karki, Navneeth Jayaprakash, Angel Pratapsingh Chavan, James Joseph Rajan

**Affiliations:** 1 Department of General Medicine, Sri Devaraj Urs Medical College, Tamaka, Kolar-563103, Karnataka, India; 2 Department of General Medicine, Malla Reddy Institute of Medical Sciences, Hyderabad, Telangana - 500 055, India; 3 Department of General Medicine, Bhaskar Medical College, Hyderabad, Telangana-500075, India; 4 Department of Medicine, Manipal College Of Medical Sciences, Fulbari, Pokhara - 11, Kaski 33700, Nepal, India; 5 Department of Medicine, D. Y. Patil Medical College Kolhapur, Maharashtra 416005, India; 6 Department of Public Health, Primary Health Centre, Chandori, Niphad, Nashik, Maharashtra, India; 7 Department of Internal Medicine, Gleneagles Healthcity Hospitals, Chennai, Tamil Nadu-600100, India

**Keywords:** Sleep apnea, left ventricular remodeling, echocardiography, cardiovascular dysfunction, apnea-hypopnea index, systolic dysfunction, diastolic dysfunction

## Abstract

Sleep apnea is a common but often overlooked condition strongly associated with cardiovascular dysfunction, particularly left ventricular (LV) remodeling. This longitudinal study assessed changes in LV structure and function over time across sleep apnea severity groups defined by the apnea-hypopnea index (AHI). Serial echocardiographic evaluations revealed that moderate-to-severe sleep apnea was significantly correlated with increased LV mass index, systolic and diastolic dysfunction. Greater AHI severity and lack of treatment were linked to more progressive LV impairment. These results emphasize the need for early diagnosis and management of sleep apnea to reduce its long-term cardiac impact.

## Background:

Recurrent airway obstruction during nocturnal sleep, leading to sympathetic activation, cycle hypoxia and haemodynamic instability, forms integral manifestations of sleep apnoea [1]. Adverse cardiac consequences like cardiac remodeling, hypertension, arrhythmias and heart failure are intrinsically related to such pathophysiological changes [2]. Left ventricular (LV) remodeling as established by hypertrophy of myocardium, fibrosis and reduced function is one of the strongest predictors of cardiovascular morbidity and mortality [3]. Irreversibly changed morphology and function of the left ventricle are increasingly related to the severity of sleep apnoea, usually graded by an apnea-hypopnea index (AHI) [4]. Cross-sectional evidence has proven a correlation of sleep apnoea with LV remodeling, although longitudinal data following this correlation over time are available in few series [5]. Periodic echocardiographic evaluation is convenient for patients with different magnitudes of sleep apnoea, as the same provides unbelievably valuable information in terms of altered left ventricle over time [6]. Early detection of high-risk individuals and early treatment with suitable remedies like CPAP therapy to prevent deleterious cardiac consequences depends on knowledge of the long-term effects [7]. Therefore, it is of interest to assess the long-term impact of severe sleep apnoea on LV remodelling, focusing especially on the changes in diastolic function, ejection fraction and LV mass index. This will help improve risk stratification and management in cardiovascular disease due to sleep apnoea.

## Methodology:

For the current longitudinal investigation, participants with sleep apnea were classified into mild, moderate and severe groups based on the apnea-hypopnea index (AHI), which was determined using nocturnal polysomnography. The presence of structural cardiac disease that would have excluded enrollment and a positive identification of obstructive sleep apnoea (OSA) were the requirements to be included in the trial. Cardiology and sleep clinics were the source of the study participants. Those with a history of myocardial infarction, valvular heart disease, or uncontrolled hypertension were excluded. A comprehensive echocardiographic study was conducted as part of the baseline clinical evaluation to determine the diastolic function, ejection fraction and left ventricular mass index after demographic data, medical history and risk factor classification had been obtained. For tracking longitudinal changes in left ventricular structure and function, serial echocardiograms at six-month intervals were done for two years. To know whether CPAP compliance was influencing the result of LV remodeling, information on CPAP compliance was ascertained. Statistical analysis comprised repeated measures ANOVA and multivariate models of regression to determine correlations among severity of sleep apnea, usage of CPAP and LV remodeling parameters. A p-value of < 0.05 was considered statistically significant. Institutional ethics committee approval was sought for the study and written informed consent was obtained from all participants.

## Results:

This research assessed the effect of the severity of sleep apnea on left ventricular remodeling during a two-year follow-up, evaluating variations in LV mass index, ejection fraction and diastolic function. Table 1 (see PDF) Baseline Characteristics of the study population, divided according to sleep apnea severity, show that patients with severe sleep apnea presented with increased BMI, hypertension and prevalence of diabetes in comparison to mild and moderate conditions. Table 2 (see PDF) Baseline Echocardiographic Parameters measurements reveal a progressive increase in LV mass index and worsening diastolic function with increasing sleep apnea severity (p<0.05). Table 3 (see PDF) LV mass index progressively increased across all groups, with the most significant rise in patients with severe sleep apnea (p<0.05). Table 4 (see PDF) Ejection fraction declined more in patients with moderate and severe sleep apnea compared to those with mild apnea, suggesting progressive LV systolic dysfunction over time (p<0.05). Table 5 (see PDF) Diastolic function worsened significantly in moderate and severe apnea patients, with a decline in the E/A ratio indicating diastolic dysfunction (p<0.05). Table 6 (see PDF) Patients using CPAP therapy showed significantly less LV remodeling, with lower LV mass index and better ejection fraction compared to non-users (p<0.05). This study assessed the impact of sleep apnea severity on left ventricular (LV) remodeling over a two-year follow-up period. Table 1 (see PDF) showed that patients with severe sleep apnea had significantly higher BMI, hypertension and diabetes prevalence, with a progressive increase in apnea severity based on the apnea-hypopnea index (AHI). Table 2 (see PDF) revealed that LV mass index, left atrial volume index and diastolic dysfunction (E/A ratio) worsened with increasing sleep apnea severity. Table 3 (see PDF) demonstrated a steady increase across all groups, with severe sleep apnea patients experiencing the most significant progression. Table 4 (see PDF) shows the similar ejection fraction declined over time, particularly in moderate and severe apnea cases, suggesting worsening systolic function. Table 5 (see PDF) shows that the Diastolic function progressively deteriorated, with a significant reduction in the E/A ratio over two years, indicating an increasing burden of diastolic dysfunction. Table 6 (see PDF) highlights CPAP therapy played a protective role as CPAP users exhibited lower LV mass index, better ejection fraction and improved diastolic function compared to non-users at 24 months. These findings emphasize the strong association between sleep apnea severity and adverse LV remodeling while highlighting the benefits of CPAP therapy in mitigating long-term cardiac dysfunction.

## Discussion:

This study demonstrates a clear association between sleep apnea severity and progressive left ventricular (LV) remodeling, characterized by increased LV mass index, declining ejection fraction and worsening diastolic function over two years [8]. Patients with moderate to severe sleep apnea exhibited significant structural and functional cardiac changes, reinforcing the role of chronic intermittent hypoxia, sympathetic over activity and hemodynamic stress in cardiac remodeling [9]. The gradual fall in ejection fraction and worsening diastolic function in the severe forms imply a greater risk of the development of heart failure, in agreement with the results of previous studies associating sleep apnea with poor cardiovascular prognosis [10]. Notably, CPAP therapy also correlated with better cardiac parameters since CPAP users had improved LV mass index and preserved ejection fraction in comparison to non-users, underlining its potential for preventing the cardiovascular effects of sleep apnea [11]. These results underscore the indication for early diagnosis and intervention in order to avert irreversible cardiac damage in those at high risk. Given the close association between AHI severity and LV remodeling, screening for subclinical cardiac dysfunction in patients with moderate to severe sleep apnea on a regular basis may be justified [12]. The results are consistent with existing data showing that structural heart damage progresses as a result of untreated sleep apnoea, underscoring the significance of focused intervention in averting long-term cardiovascular morbidity [13]. To further improve cardiac outcomes in individuals with sleep apnoea, future research must examine the long-term effects of CPAP compliance and other therapeutic approaches.

## Conclusion:

We show that LV remodelling, the advancement of systolic and diastolic dysfunction and increased cardiovascular risk are all strongly associated with the degree of sleep apnoea. The findings demonstrate the protective impact of CPAP therapy against these harmful changes, reinforcing the need of early diagnosis and intervention. By effectively treating sleep apnoea, cardiovascular outcomes can be improved and long-term structural heart damage can be prevented.
